# Supplementary material for: Effects of Exogenous Ergothioneine on Brassica rapa Clubroot Development Revealed by Transcriptomic Analysis
Source: Int J Mol Sci. 2023 Mar 28;24(7):6380. doi: 10.3390/ijms24076380 (PMC10094275; doi:10.3390/ijms24076380)
Supplement: Supplementary file 1 [file ijms-24-06380-s001.zip › Figure S1. Effects of different concentrations of EGT on germination of resting spores of P. brassicae..pdf]

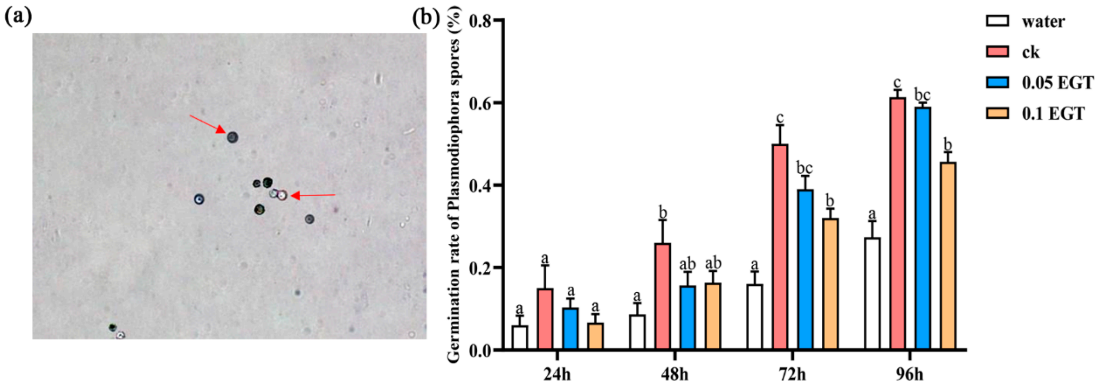


**Figure S1.** Effects of different concentrations of EGT on germination of resting spores of *P. brassicae*. (**a**) Microscopic observation of resting spores of *P. brassicae*. Under orcein staining, resting spores were stained red, while germinating spores were not stained. (**b**) Investigation on germination rate of dormant spores of *P. brassicae* at different time under different treatments. White, red, blue, and yellow bars are blank control with water, root exudates, root exudates plus 0.05 mM ergothioneine, and root excretions plus 0.1 mM ergothioneine, respectively. Black letters indicate significant differences between treatments (one-way ANOVA, $p < 0.05$).
